# Antidiabetic drug glyburide modulates depressive-like behavior comorbid with insulin resistance

**DOI:** 10.1186/s12974-017-0985-4

**Published:** 2017-10-30

**Authors:** Wen-Jun Su, Wei Peng, Hong Gong, Yun-Zi Liu, Yi Zhang, Yong-Jie Lian, Zhi-Yong Cao, Ran Wu, Lin-Lin Liu, Bo Wang, Yun-Xia Wang, Chun-Lei Jiang

**Affiliations:** 1 Laboratory of Stress Medicine, Faculty of Psychology and Mental Health, Second Military Medical University, 800 Xiangyin Road, Shanghai, China; 2 Department of Psychiatry, Faculty of Psychology and Mental Health, Second Military Medical University, 800 Xiangyin Road, Shanghai, China; 3 Department of Psychiatry, The 102nd Hospital of PLA, 55 North Heping Road, Changzhou, China

**Keywords:** Depression, Insulin resistance, Glyburide, NLRP3 inflammasome, Stress, Inflammation

## Abstract

**Background:**

Abundant reports indicated that depression was often comorbid with type 2 diabetes and even metabolic syndrome. Considering they might share common biological origins, it was tentatively attributed to the chronic cytokine-mediated inflammatory response which was induced by dysregulation of HPA axis and overactivation of innate immunity. However, the exact mechanisms remain obscure. Herein, we mainly focused on the function of the NLRP3 inflammasome to investigate this issue.

**Methods:**

Male C57BL/6 mice were subjected to 12 weeks of chronic unpredictable mild stress (CUMS), some of which were injected with glyburide or fluoxetine. After CUMS procedure, behavioral and metabolic tests were carried out. In order to evaluate the systemic inflammation associated with inflammasome activation, IL-1β and inflammasome components in hippocampi and pancreases, as well as corticosterone and IL-1β in serum were detected separately. Moreover, immunostaining was performed to assess morphologic characteristics of pancreases.

**Results:**

In the present study, we found that 12 weeks’ chronic stress resulted in depressive-like behavior comorbid with insulin resistance. Furthermore, antidiabetic drug glyburide, an inhibitor of the NLRP3 inflammasome, was discovered to be effective in preventing the experimental comorbidity. In brief, it improved behavioral performance, ameliorated insulin intolerance as well as insulin signaling in the hippocampus possibly through inhibiting NLRP3 inflammasome activation by suppressing the expression of TXNIP.

**Conclusions:**

All these evidence supported our hypothesis that chronic stress led to comorbidity of depressive-like behavior and insulin resistance via long-term mild inflammation. More importantly, based on the beneficial effects of blocking the activation of the NLRP3 inflammasome, we provided a potential therapeutic target for clinical comorbidity and a new strategy for management of both diabetes and depression.

## Background

As the pace of life to speed up, coupled with unhealthy diet habits and lifestyles, people are suffering from more complex and intense stress in both physical and psychological aspects. Long-term exposure to these hazards has been considered as a major cause of many chronic diseases [1]. It is well known that depression and diabetes, especially type 2 diabetes (T2D), were among the leading causes of global disability-adjusted life-years (DALYs) [2]. According to reports from World Health Organization, more than 300 million human beings all over the world suffer from depression (refer to URL: http://www.who.int/mediacentre/factsheets/fs369/en/), while approximately 422 million people worldwide have diabetes (refer to URL: http://www.who.int/mediacentre/factsheets/fs312/en/). In brief, depression is a devastating mental disorder characterized by anhedonia, sadness, feelings of guilt or fatigue, disrupted sleep or loss of appetite, and poor concentration. At worst, it would lead to suicides. Apart from that, diabetes is usually diagnosed by elevated plasma glucose levels, whereas abnormalities that present before glucose changes may sometimes be undetectable. More individuals are in high-risk diabetic states—prediabetes, which is associated with the co-occurrence of insulin resistance (IR) and β-cell dysfunction [3].

Surprisingly, it was discovered that depression, T2D, and even metabolic syndrome (MetS) are often comorbid. T2D was associated with a higher susceptibility to depression [4], while depression increased T2D risk by 60% [5–7]. Moreover, this bidirectional association cannot be attributed to the usage of antidepressants [8]. Recently, abundant evidence hinted that depression and T2D might share biological origins. Researchers postulated that the underlying mechanism could be the chronic cytokine-mediated inflammatory response which was induced by dysregulation of hypothalamic-pituitary-adrenal (HPA) axis and overactivation of innate immunity [9–11].

In fact, this hypothesis was based on various clinical and preclinical evidence. Among them, we found out some inspirational clues. A multitude of researches indicated that activation of NLRP3 (nucleotide-binding domain, leucine-rich-containing family, pyrindomain-containing-3) inflammasome and subsequent production of Interleukin-1β (IL-1β) contribute to various metabolic abnormalities, particularly IR-related T2D and MetS. It was demonstrated that ablation of genes of inflammasome components and IL-1β could protected mice from fatty acid-induced IR [12–14]. Treating rodent diabetic models with anti-IL-1β therapy resulted in ameliorated insulin insensitivity as well as improved β-cell survival [15]. Furthermore, Lee et al. [16] discovered overactivation of the NLRP3 inflammasome in type 2 diabetic inpatients. Prior to that, Larsen [17] and colleagues found that anakinra, a blockade of IL-1, improved glycemia and β-cell secretory function in patients with T2D. Hence, NLRP3 inflammasome was considered a bond between chronic inflammation and metabolism.

In addition, it seems that NLRP3 inflammasome and IL-1β also play pivotal roles in depression [18]. According to Goshen et al. [19], cerebral IL-1 mediated stress-induced depressive-like behavior in mice via suppressing hippocampal neurogenesis and activating HPA axis. Updated studies on this issue, including that from our laboratory, proved that depressive-like behaviors induced by CUMS required a functional NLRP3 inflammasome [20–22]. Moreover, clinical evidence showed that NLRP3 inflammasomes in mononuclear blood cells of depressive patients were activated [23]. Besides, depression was accompanied by an increase in pro-inflammatory cytokine levels including IL-1, while associated with the alleviation of immune overactivity when symptoms relived [24]. Therefore, scholars across the world postulated that therapies targeting the NLRP3 inflammasome might be a novel strategy for depression [25, 26].

Collectively, NLRP3 inflammasome activation and subsequent IL-1β generation were involved in the development of depression and T2D separately. We tentatively presume that this common pathogenesis might be the “shared biological origin.” That is to say, chronic hyperactivation of HPA axis and consequent overreaction of innate immunity were blamed for the comorbidity [11, 27]. For instance, glucocorticoids (GC) could supervise immune system and prime the NLRP3 inflammasome [28, 29], as well as induce apoptosis in β-cells via thioredoxin-interacting protein (TXNIP) [30] and instigate IR through pathways including prompting immune reactions [1, 31]. Additionally, it was reported that anti-diabetic drug glyburide was an effective inhibitor of NLRP3 inflammasome [32, 33], while fluoxetine could also improve hyperglycemia [34]. Herein, we designed this study to investigate the comorbidity of depression and T2D/IR, as well as unravel possible molecular mechanisms.

## Methods

### Animals and drugs

Male 6-week-old C57BL/6 mice were introduced from Experimental Animal Center of Second Military Medical University (Shanghai, China). All the animals were bred in a standardized animal room (temperature 22 ± 2 °C, lights on during 7 a.m.–7 p.m.), with free access to clean tap water and rodent chow. For acclimation, mice were treated with 1% sucrose solution (weight/volume) for 14 days according to our previous study [21]. Thereafter, the rodents were randomly allocated to 5 independent groups, namely Control, CUMS, CUMS + Vehicle (Veh), CUMS + Glyburide (Glb) and CUMS + Fluoxetine (Flx) (*n* = 8). Drugs used within the procedure were dissolved in 2% DMSO and 2% Tween-80 (Sigma-Aldrich, St. Louis, MO, USA) normal saline, then injected at the dose of 10 mg/kg/day intraperitoneally (i.p.) according to former studies [32, 35–38]. Before each injection, drug solutions will be mixed thoroughly and heated to 37 °C. Glyburide was purchased from Sigma-Aldrich (#G0639, St. Louis, MO, USA) while fluoxetine hydrochloride was bought from MedChem Express (#HY-B0102A, Shanghai, China).

### Chronic unpredictable mild stress protocol

The chronic unpredictable mild stress (CUMS) paradigm was adapted from our past research [21, 39]. Briefly, it lasted for 12 weeks and contained diverse randomly assigned stressors: swimming at 4 °C for 5 min; 45 °C dry-heat stress for 10 min; cage vibration for 30 min; constraint for 2 h; 45° cage inclination for 12 h; damp bedding for 16 h; continuous illumination for 24 h; food or water deprivation for 24 h. During the stress procedure, only one stressor was performed per day, and no single stressor was conducted consecutively for 2 days. Animals that were subjected to CUMS were kept separately. Drug administrations were maintained throughout the whole CUMS procedure. Baseline data including specific behavioral and glucose metabolic parameters were acquired before and after the protocol.

### Behavioral tests

The behavioral tests were all carried out in dark phase (18:00–22:00 p.m.). To eliminate olfactory interference, the trial chambers were wiped with 75% ethanol between two separated test sessions in tail suspension tests and open field tests, respectively.

#### Sucrose preference test

Sucrose preference test (SPT) is a widely used method of evaluating depressive-like behavior in animals. Generally, it represents anhedonia, one of the major symptoms of depression in human beings. According to literature, food and water were deprived 20 h before SPT [21]. During the test, each animal would be provided with two bottles of the same size: one containing clean tap water and the other containing 1% sucrose solution. Fluid consumptions were checked after the 1-h test. Sucrose preference proportion = sucrose solution consumption/(sucrose solution consumption + tap water consumption) *100%.

#### Tail suspension test

Tail suspension test (TST) is another common behavioral test, which reflected the state of despair and helplessness. At the end of CUMS protocol, the mouse would be hung upside down on a hook by the tail in the PHM-300 tail suspension chamber (MED Associates Inc., St. Albans, VT, USA). After 1 min of adaptation to the apparatus, the immobility durations during the next 5 min’ test were recorded and analyzed with the TST software (lower threshold value = 0.25).

#### Open field test

Open field test (OFT) was used to measure spontaneous activity, aiming at identifying anxious and sickness behavior. It was generally believed that total traveling distance mainly reflected sickness behavior of animals, whereas time spent in central and periphery areas would indicate anxious status. During the tests, the mice were carefully placed in the middle of trial chambers, consisting of perforated acrylonitrile butadiene styrene copolymers (#RD1112-IOF, Mobile Datum Information Technology, Shanghai, China). The 5-min movement traces of the mice were recorded by infrared cameras and analyzed by OFT software.

### Metabolic measurements

#### Intraperitoneal glucose tolerance test

For the glucose tolerance tests (GTT), mice were fasted overnight (16 h) in clean homecages and then injected (i.p.) with 2 g/kg body weight of glucose solution. Blood samples for glucose measurements were collected gently from the tail veins and dropped immediately onto glucometer strips. MAJOR II Blood Glucose Monitoring System (Major Biosystem, Taiwan, China) were utilized for fast blood-glucose detection at 0, 30, 60, 90, and 120 min after glucose injection. The calculation of areas under curve (AUC) was based upon the polygonal lines joining glucose values for different time sections.

#### Intraperitoneal insulin tolerance test

For the insulin tolerance tests (ITT), mice were administrated (i.p.) with 1 IU/kg body weight of Novolin R (Novo Nordisk, Bagsvaerd, Denmark) after 6 h’ fasting. Same as GTT, blood samples were acquired from the tail veins and measured immediately. AUCs were calculated according to the connecting lines of blood glucose values at 0, 30, 60, 90, and 120 min after insulin injection.

#### Glucose-stimulated insulin secretion test in vivo

Mice were starved for 16 h and then injected (i.p.) with 1 mg/kg of glucose solution. Blood was collected softly from the tail veins using Microvette® CB 300 Lithium Heparin tubes (Sarstedt, Nümbrecht, Germany) at 0 and 30 min after glucose injection. The plasma samples were separated in a centrifuge at 2000*g* for 5 min. The determination of plasma insulin would be conducted as described below. The homeostasis model of assessment for insulin resistance index (HOMA-IR) values were calculated according to a previous study [40]: [fasting blood glucose (mg/dl)* fasting plasma insulin (μU/ml) /405]. Plasma insulin (μU/ml) was determined using titer of human insulin (26 U/mg), for that of mice was not defined uniformly.

### Sacrifice and sample preparation

After completion of behavioral and metabolic tests, mice were sacrificed under general anesthesia with pentobarbital. Blood was collected, followed by coagulation at room temperature for 30 min and then centrifuged at 4000 rpm for 15 min. The supernatant serum was separated and preserved at −80 °C. Hippocampi were dissected and flash frozen in liquid nitrogen immediately after decapitation. Pancreases were cut into two parts along their long axes, one fixed with cold 4% paraformaldehyde and the other flash frozen and stored at −80 °C. Serum samples would be used for the detection of Corticosterone and Interleukin-1β, hippocampi and frozen pancreas were utilized in Western Blotting, whereas fixed pancreases were applied for immunostaining.

### Enzyme-linked immunosorbent assays

Serum corticosterone and IL-1β levels were measured by Mouse Corticosterone ELISA kit (#F10246, Westang, Shanghai, China) and Mouse IL-1beta Platinum ELISA kit (#BMS6002, eBioscience, San Diego, CA, USA) according to the manufacturer’s instructions. Plasma insulin levels were determined by Ultra Sensitive Mouse Insulin ELISA Kit (#90080, Crystal Chem, Downers Grove, IL, USA) following the manufacturer’s protocol.

### Western Blotting

The hippocampi and pancreases were homogenized in the ice-cold RIPA lysis buffer (#P0013B, Beyotime Biotechnology, Nantong, Jiangsu, China) with protease inhibitor cocktail (#B14001, Bimake, Shanghai, China) and PhosSTOP (#04906845001, Roche, Indianapolis, IN, USA) as previously described [21]. The protein concentration was determined by Enhanced BCA Protein Assay Kit (#P0010, Beyotime Biotechnology, Nantong, Jiangsu, China). The centrifuged lysates were added with 5X loading buffer (#P0015, Beyotime Biotechnology, Nantong, Jiangsu, China) at 4:1 volume ratio, vortexed and then boiled for 10 min. Equal amounts of protein samples were loaded and separated on 10% or 12% SDS-PAGE gels (#P0012A, Beyotime Biotechnology, Nantong, Jiangsu, China). After electrophoresis, proteins were transferred onto Immobilon-P PVDF membranes (Millipore, Billerica, MA, USA) using a Bio-Rad (Hercules, CA, USA) wet transfer system. Afterwards, the blots were blocked with 0.1% Tween-20 solution (TBS-T) containing 3% bovine serum albumin (BSA) and incubated with appropriate primary antibodies and then specific IRDye conjugated secondary antibodies. Finally, the membranes were scanned, and the intensities of protein bands were quantified using Odyssey Infrared Imaging System (LI-COR, Inc., Lincoln, NE, USA) and Image J Software (NIH, Bethesda, MD, USA). Resultant values were normalized to grayscale intensities of the total (non-phosphorylated) protein levels and that of internal reference GAPDH to reduce inter- and intra-gel variability. Analyzed data were displayed as fold change vs. Control levels.

Primary antibodies for NLRP3 (#ab4207), IL-1β (#ab9722), and TXNIP (#ab188865) were bought from Abcam plc. (Cambridge, UK). Primary antibodies for Caspase-1 p10 (#sc-514), IRS1 (#sc-559) and phospho-IRS1^Ser307^ (#sc-33,956) were purchased from Santa Cruz Biotechnology (Santa Cruz, CA, USA). Rabbit anti-mouse primary antibodies for Akt (#1080–1) and phospho-Akt^Ser473^ (#2118–1) were bought from Epitomics (Burlingame, CA, USA). Rabbit anti-mouse GAPDH antibody (#D110016) was purchased from Sangon Biotech (Shanghai, China). IRDye secondary antibodies were introduced from LI-COR Biosciences (LI-COR, Inc., Lincoln, NE, USA).

### Immunostaining and morphometric analysis

Paraffin-embedded pancreatic sections were incubated with specific primary and secondary antibodies, then observed under Axiovision Observer A1 fluorescence microscope (Carl Zeiss, Germany) and photographed using the accompanying software. In accordance with former researches [38, 41], quantifications of insulin, glucagon and F4/80 positive area proportions per islet were performed using the manual histology tools in Image J Software (NIH, Bethesda, MD, USA). Four pairs of 4-μm-thick serial sections, 100 μm apart were analyzed. An average of 53 islets from 3 to 4 mice for each group were analyzed for insulin and glucagon areas, and 67 islets from 6 mice per group were assessed for F4/80 areas.

Primary antibodies for Insulin (#GB13121), Glucagon (#GB13097), F4/80 (#GB11027) and Cy3/Alexa 488 conjugated fluorescent secondary antibodies (#GB21301/#GB25303) were all purchased from Servicebio Biotechnology (Wuhan, Hubei, China). Fluorescent stain solution 4′, 6-diamidino-2-phenylindole dihydrochloride (DAPI) for nuclei labeling was bought from the same company.

### Statistical analysis

The data were expressed as mean ± standard error (SEM) and differences were defined statistically significant only when *p* < 0.05. Data were mainly analyzed using one-way analysis of variance (ANOVA) followed by Bonferroni’s multiple comparison post hoc tests with GraphPad Prism 6 (GraphPad Software, Inc., La Jolla, CA, USA). Two-way ANOVAs for repeated measurements followed by Tukey’s or Bonferroni’s post hoc tests were used in the analyses of GSIS data and time section values of GTT and ITT.

## Results

### Glyburide abrogated depressive-like behavior induced by CUMS

**Fig. 1:**
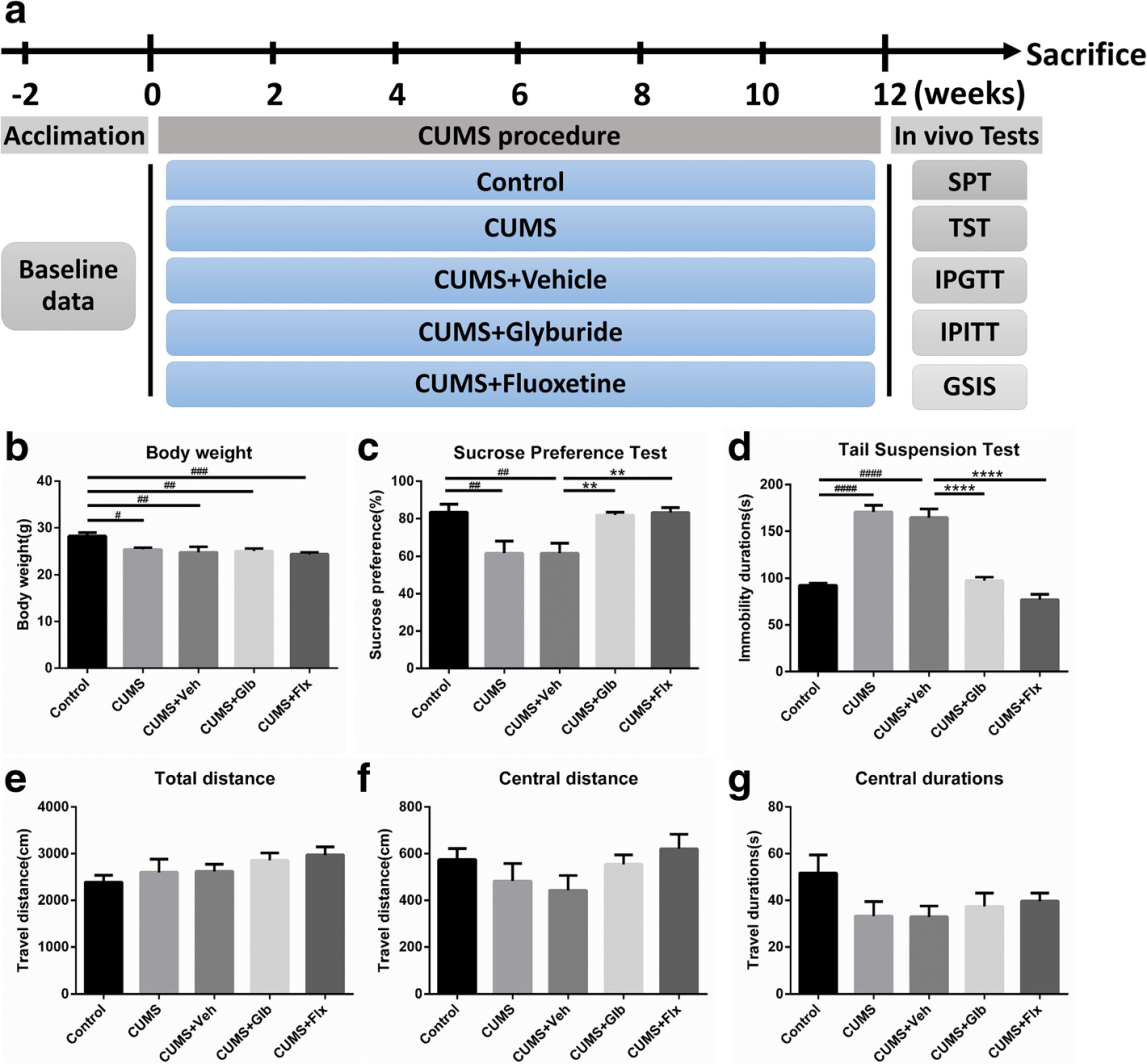
Chronic unpredictable mild stress (CUMS) schedule and behavioral analysis after 12 weeks. Drug treatment sustained 1/day throughout the whole CUMS procedure (**a**). All stressed mice showed noticeably fewer body weight gains (**b**) (^#^ *p* < 0.05, ^##^ *p* < 0.01, ^###^ *p* < 0.001 vs. Control). Mice in CUMS and CUMS + Veh groups displayed declined sucrose preference (**c**) and increased tail suspension immobility durations (**d**) (^##^ *p* < 0.01, ^####^ *p* < 0.0001 vs. Control), while there was no significant difference among those parameters in the open field test (**e**–**g**). Compared with CUMS + Veh, mice of CUMS + Glb and CUMS + Flx got improved sucrose preference (**c**) and decreased immobility time (**d**). (^**^ *p* < 0.01, ^****^ *p* < 0.0001 vs. CUMS + Veh). Data presented as mean ± SEM, *n* = 6~8

After acclimation and group assignment, the experimental mice were subjected to CUMS and drug administration simultaneously throughout the 12 weeks’ CUMS procedure (Fig. 1a). Comparing with the non-stressed control mice, all the mice underwent CUMS showed lighter body weight (respectively, *t*_(31)_ = 2.914, *p* < 0.05; *t*_(31)_ = 3.535, *p* < 0.01; *t*_(31)_ = 3.545, *p* < 0.01; *t*_(31)_ = 4.326, *p* < 0.001. Fig. 1b). To determine the potential antidepressive effect of glyburide, behavioral analyses were performed. As compared to Control, both CUMS and CUMS + Veh mice showed fewer percentages of sucrose preference (respectively, *t*_(31)_ = 3.667, *p* < 0.01; *t*_(31)_ = 3.661, *p* < 0.01. Figure 1c) and longer durations of tail suspension immobility (respectively, *t*_(31)_ = 9.567, *p* < 0.0001; *t*_(31)_ = 8.829, *p* < 0.0001. Fig. 1d). However, in comparison with CUMS + Veh, sucrose preference levels were higher in both CUMS + Glb (*t*_(31)_ = 3.402, *p* < 0.01) and CUMS + Flx (*t*_(31)_ = 3.620, *p* < 0.01) groups (Fig. 1c). Similarly, shorter immobility time was presented in CUMS + Glb (*t*_(31)_ = 8.241, *p* < 0.0001) and CUMS + Flx (*t*_(31)_ = 10.720, *p* < 0.0001) groups when comparing to CUMS + Veh (Fig. 1d). Meanwhile, no significant differences were detected in results of open field, including total travel distance, central travel distance and time spent in central areas (Fig. 1e–g).

### Glyburide modulated insulin signaling disturbance induced by CUMS

**Fig. 2:**
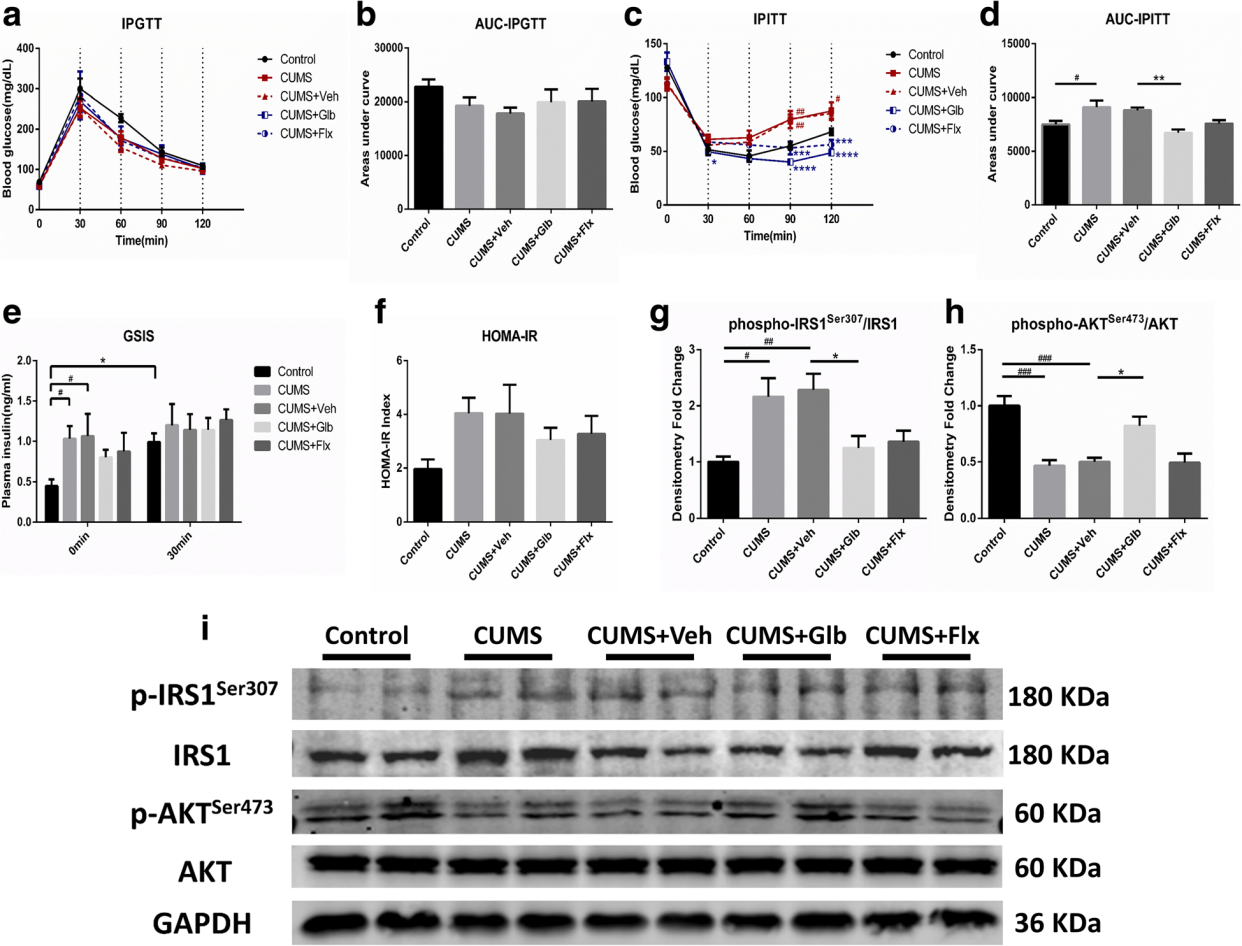
Detection of glucose metabolic parameters and hippocampal insulin signaling pathway. No statistical difference was discovered among the five groups in IPGTT results (**a, b**). As for ITT, it presented that CUMS mice got larger AUC than that of the control group, while the AUC of CUMS + Glb group was smaller as compared to that of CUMS + Veh (**c, d**) (^#^ *p* < 0.05 vs. Control, ^**^ *p* < 0.01 vs. CUMS + Veh). In GSIS test, it showed that before the injection of glucose, CUMS and CUMS + Veh mice got higher levels of plasma insulin; besides, 30 min after glucose administration, non-stressed control but not stressed mice showed a significant increase of plasma insulin (**e**) (^#^ *p* < 0.05, ^*^ *p* < 0.05). Although not significantly enough, there existed a rising trend of HOMA-IR index in stressed mice, especially that of CUMS and CUMS + Veh groups as compared with Control mice (**f**). In comparison, CUMS and CUMS + Veh mice presented more phosphorylation of IRS(Ser307) and less phosphorylation of AKT(Ser473) in hippocampi than that of Control, remarkably, CUMS + Glb reversed those changes meaningfully (**g**–**i**) (^#^ *p* < 0.05, ^##^ *p* < 0.01, ^###^ *p* < 0.001 vs. Control, ^*^ *p* < 0.05 vs. CUMS + Veh). Data presented as mean ± SEM, *n* = 6 for **a**–**f**, *n* = 4 for **g**–**i**

In order to evaluate the effect of CUMS on glucose metabolism and insulin signaling, metabolic measurements in vivo, including GTT, ITT, and GSIS, were conducted at the end of the CUMS protocol. Generally, there was no significant difference in GTT among distinct groups (Fig. 2a, b). As was presented, CUMS group got larger AUC in ITT than that of Control (*t*_(25)_ = 2.852, *p* < 0.05), whereas the AUC of CUMS + Glb was not as large as CUMS + Veh (*t*_(25)_ = 3.770, *p* < 0.01) (Fig. 2c, d). Moreover, in GSIS test, it could be easily discovered that in fasting condition, plasma insulin concentrations of both CUMS and CUMS + Veh mice were higher than that of Control (respectively, *q*_(20)_ = 4.415, *p* < 0.05; *q*_(20)_ = 4.653, *p* < 0.05). After glucose injection, insulin levels in Control raised up significantly (*t*_(20)_ = 2.895, *p* < 0.05), while the other groups did not demonstrate such an increase (Fig. 2e). Based on the results of fasting blood glucose and plasma insulin, HOMA-IR indexes were determined according to a particular formula. Though not statistically significant, there did exist an ascending tendency in stressed mice, especially CUMS and CUMS + Veh groups, compared with Control (Fig. 2f). To further investigate the insulin signaling pathway in the hippocampus, phosphorylations of IRS1^Ser307^ and Akt^Ser473^ were detected using western blots. As compared with Control, mice of CUMS and CUMS + Veh got enhanced phosphorylation of IRS1^Ser307^ (respectively, (*t*_(15)_ = 3.415, *p* < 0.05; (*t*_(15)_ = 3.769, *p* < 0.01) and downregulated of p-Akt^Ser473^ (respectively, (*t*_(15)_ = 5.392, *p* < 0.001; (*t*_(15)_ = 5.040, *p* < 0.001), injection of glyburide could prevent those changes (p-IRS^Ser307^ *t*_(15)_ = 3.037, *p* < 0.05; p-Akt^Ser473^ *t*_(15)_ = 3.246, *p* < 0.05). Interestingly, it seemed that fluoxetine also had the potential to modulate IRS phosphorylation, even though not significantly (Fig. 2g–i).

### Glyburide and fluoxetine inhibited NLRP3 inflammasome activation in hippocampi via downregulating TXNIP

**Fig. 3:**
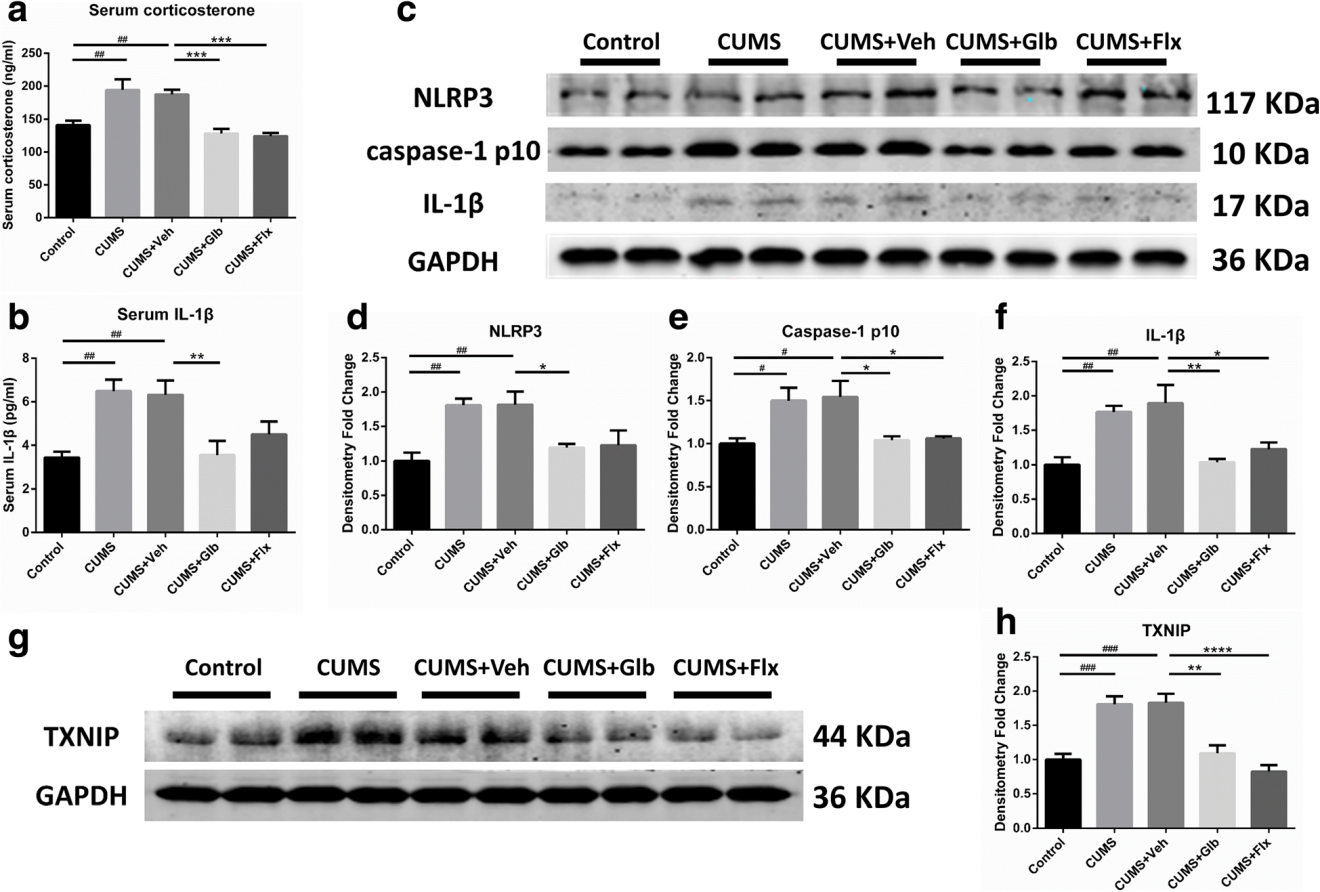
Detection of NLRP3 inflammasome activation related molecules in serum and hippocampi. Compared to Control, mice in CUMS and CUMS + Veh groups had higher concentrations of corticosterone and IL-1β (^##^ *p* < 0.01 vs. Control), while such increases were nearly normalized in both CUMS + Glb and CUMS + Flx (^**^ *p* < 0.01, ^***^ *p* < 0.001 vs. CUMS + Veh) (**a, b**). In addition, mice from CUMS and CUMS + Veh groups also got higher protein levels of hippocampal NLRP3, caspase-1 p10 and IL-1β than that of Control (^#^ *p* < 0.05, ^##^ *p* < 0.01 vs. Control). As comparing with CUMS + Veh, caspase-1 p10 and IL-1β levels of CUMS + Glb and CUMS + Flx mice were significantly lower. Moreover, CUMS + Glb also contained less NLRP3 protein (^*^ *p* < 0.05, ^**^ *p* < 0.01 vs. CUMS + Veh) (**c**–**f**). Besides, TXNIP strikingly increased in CUMS and CUMS + Veh groups (^###^ *p* < 0.001 vs. Control), but when comparing with CUMS + Veh, it decreased sharply in both CUMS + Glb and CUMS + Flx groups (^**^ *p* < 0.01, ^****^ *p* < 0.0001 vs. CUMS + Veh) (**g**, **h**). Data presented as mean ± SEM, *n* = 6 for **a, b**, *n* = 4 for **c**–**h**

Chronic stress enhanced secretions of corticosterone and IL-1β, which was indicated in the comparison of CUMS (respectively, *t* (25) = 4.079, *p* < 0.01; t (25) = 3.863, *p* < 0.01) and CUMS + Veh (respectively, *t* (25) = 3.579, *p* < 0.01; *t* (25) = 3.641, *p* < 0.01) with Control. However, injection of glyburide could normalize serum corticosterone (*t* (25) = 4.577, *p* < 0.001) and IL-1β (*t* (25) = 3.491, *p* < 0.01) when comparing with CUMS + Veh. The functions of fluoxetine were similar with glyburide in regulating serum corticosterone (*t* (25) = 4.872, *p* < 0.001) and IL-1β (Fig. 3a, b). To determine the activation of the NLRP3 inflammasome, IL-1β and inflammasome components in hippocampi were detected. As was displayed, relative protein levels of IL-1β, caspase-1 p10 and NLRP3 were significantly upregulated in both CUMS (respectively, t _(15)_ = 3.762, *p* < 0.01; t _(15)_ = 3.112, *p* < 0.05; t (15) = 3.834, *p* < 0.01) and CUMS + Veh (respectively, t (15) = 4.395, p < 0.01; t _(15)_ = 3.379, _*p*_ < 0.05; t _(15)_ = 3.878, *p* < 0.01) groups. Nevertheless, glyburide (IL-1β *t*_(15)_ = 4.218, *p* < 0.01; p10 *t*_(15)_ = 3.144, *p* < 0.05; NLRP3 *t*_(15)_ = 2.982, *p* < 0.05) and fluoxetine (IL-1β *t*_(15)_ = 3.290, *p* < 0.05; p10 *t*_(15)_ = 3.010, *p* < 0.05) hindered those upregulations (Fig. 3c–f). Apart from that, inspections on upstream signaling of NLRP3 inflammasome was performed. Expression of TXNIP was enhanced in CUMS (t _(15)_ = 5.268, *p* < 0.001) and CUMS + Veh (*t*_(15)_ = 5.407, *p* < 0.001) groups versus Control, but impeded in CUMS + Glb (t _(15)_ = 4.803, *p* < 0.01) and CUMS + Flx (*t*_(15)_ = 6.551, *p* < 0.0001) groups versus CUMS + Veh (Fig. 3g, h).

### CUMS led to shrinkage of islet insulin-positive areas and infiltration of F4/80-positive cells

**Fig. 4:**
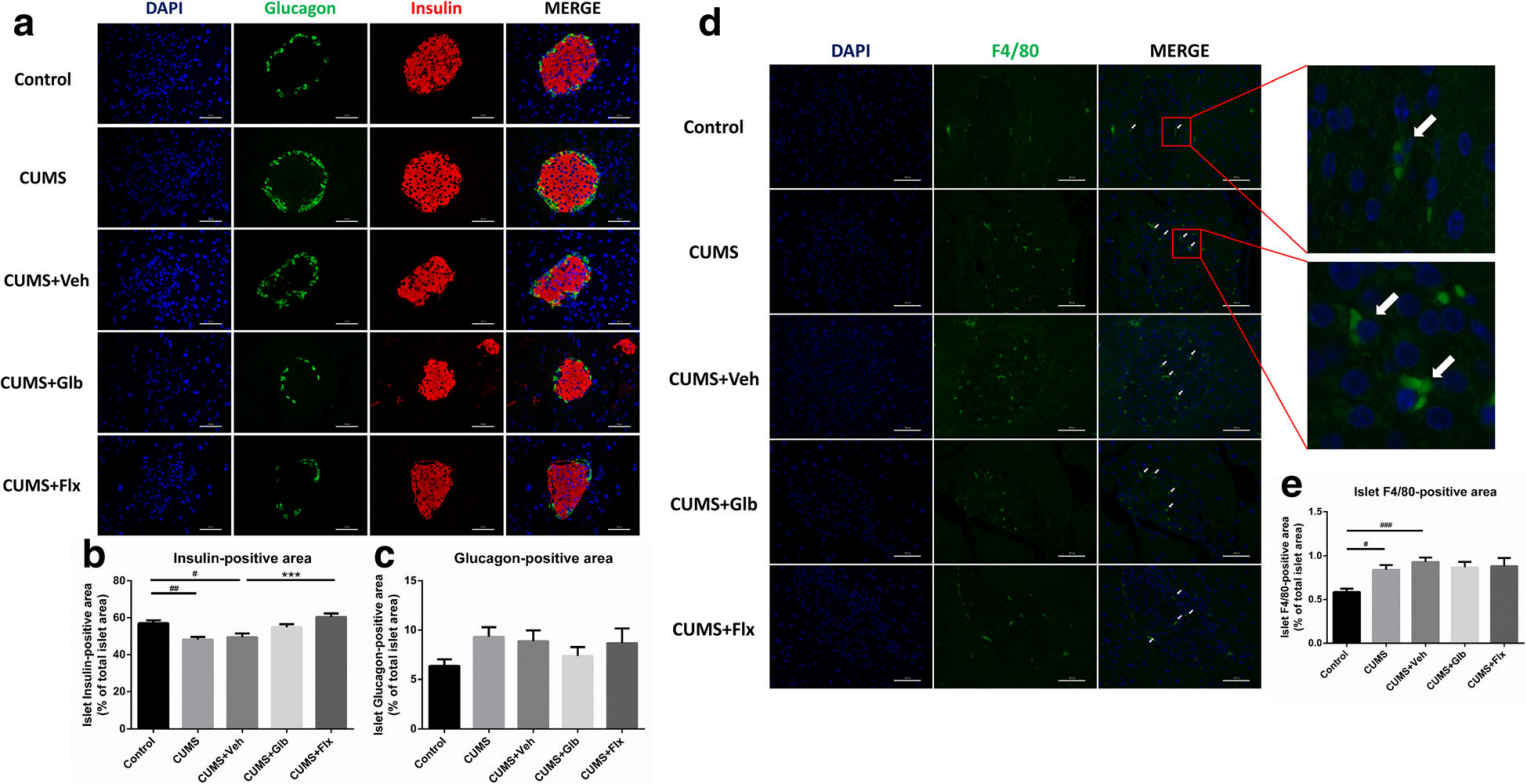
Morphometric analysis of Insulin-, Glucagon- and F4/80-positive cells in pancreatic islets. Pancreatic islet Insulin-positive area proportion was lower in both CUMS and CUMS + Veh groups than that of Control group (^#^ *p* < 0.05, ^##^ *p* < 0.01 vs. Control), while it was higher in CUMS + Flx than that of CUMS + Veh (^***^ *p* < 0.001 vs. CUMS + Veh) (**a**–**c**). Meanwhile, mice of CUMS and CUMS + Veh got a higher percentage of islet F4/80-positive area than Control (^#^ *p* < 0.05, ^###^ *p* < 0.001 vs. Control) (**d, e**). Immunofluorescent pictures were taken under 400X magnification, scale bars = 50 μm, white arrows in D denoted F4/80 immunostaining positive. Data presented as mean ± SEM, *n* = 3~4 for **a**–**c**, *n* = 6 for **d, e**

According to immunostaining and morphometric analysis, insulin-positive/islet areas significantly decreased in CUMS (*t*_(258)_ = 3.602, *p* < 0.01) and CUMS + Veh (*t*_(258)_ = 2.824, *p* < 0.05) vs. Control group, while CUMS + Flx (*t*_(258)_ = 4.219, *p* < 0.001) elevated the proportion vs. CUMS + Veh (Fig. 4a, b). Glucagon-positive areas per islet were not different among separate groups (Fig. 4a, c). Surprisingly, compared with islets from mice of Control, F4/80-positive/islet areas were increased in that of CUMS (*t*_(329)_ = 2.744, *p* < 0.05) and CUMS + Veh (*t*_(329)_ = 3.932, *p* < 0.001) (Fig. 4d, e).

### Glyburide and fluoxetine impeded activation of NLRP3 inflammasome in pancreas by suppressing upstream molecule TXNIP

**Fig. 5:**
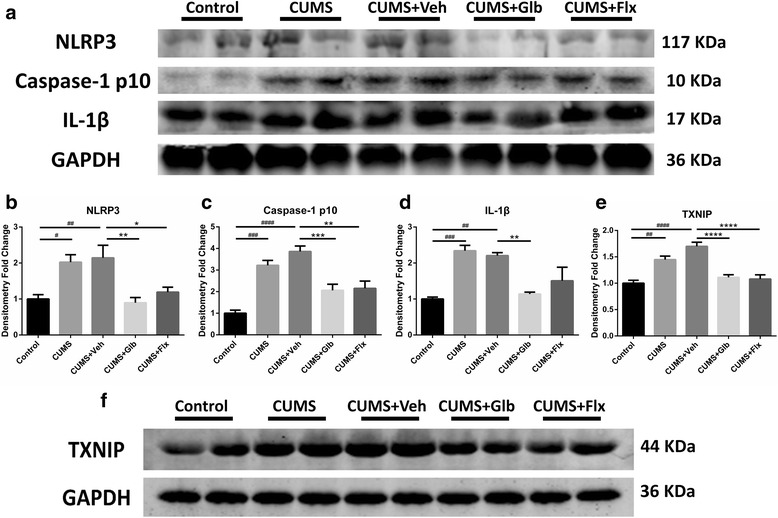
Detection of NLRP3 inflammasome components and upstream molecule TXNIP in pancreas. Compared to Control, mice in CUMS and CUMS + Veh groups got higher protein levels of pancreatic NLRP3, caspase-1 p10 and IL-1β than that of Control (^#^ *p* < 0.05, ^##^ *p* < 0.01, ^###^ *p* < 0.001, ^####^ *p* < 0.0001 vs. Control). As comparing with CUMS + Veh, NLRP3 and caspase-1 p10 levels of CUMS + Glb and CUMS + Flx mice were significantly lower, moreover, CUMS + Glb also got less IL-1β (^*^ *p* < 0.05, ^**^ *p* < 0.01, ^***^ *p* < 0.001, ^****^ *p* < 0.0001 vs. CUMS + Veh) (**a**–**d**). Besides, TXNIP was upregulated in CUMS and CUMS + Veh groups (^##^ *p* < 0.01, ^####^ *p* < 0.0001 vs. Control), but when comparing with CUMS + Veh, it downregulated prominently in both CUMS + Glb and CUMS + Flx groups (^****^ *p* < 0.0001 vs. CUMS + Veh) (**e, f**). Data represent mean ± SEM, *n* = 4

Expression of inflammasome components NLRP3 and caspase-1 p10 were promoted in the pancreas of mice from CUMS (respectively *t*_(15)_ = 3.450, *p* < 0.05; *t*_(15)_ = 6.100, p < 0.001) and CUMS + Veh (*t*_(15)_ = 3.860, *p* < 0.01; *t*_(15)_ = 7.866, *p* < 0.0001) versus that of Control (Fig. 5a–c). Furthermore, protein levels of IL-1β were elevated in CUMS (*t*_(15)_ = 5.034, *p* < 0.001) and CUMS + Veh (*t*_(15)_ = 4.528, *p* < 0.01) (Fig. 5a, d). Yet, as compared to CUMS + Veh group, fluoxetine downregulated levels of NLRP3 (*t*_(15)_ = 3.213, *p* < 0.05) and caspase-1 p10 (*t*_(15)_ = 4.695, *p* < 0.01), just like glyburide prohibited the upregulation of NLRP3 (*t*_(15)_ = 4.206, *p* < 0.01), p10 (*t*_(15)_ = 4.948, *p* < 0.001) and IL-1β (*t*_(15)_ = 4.003, *p* < 0.01) (Fig. 5a–d). Similar to alterations in hippocampi, expressions of pancreatic TXNIP were enhanced in CUMS (*t*_(15)_ = 4.693, *p* < 0.01) and CUMS + Veh (*t*_(15)_ = 7.328, *p* < 0.0001) groups vs. Control; but when comparing with CUMS + Veh, it decreased apparently in both CUMS + Glb (*t*_(15)_ = 6.174, *p* < 0.0001) and CUMS + Flx (*t*_(15)_ = 6.502, *p* < 0.0001) group (Fig. 5e, f).

## Discussion

In the present study, we found that depressive-like behavior co-occurred with abnormalities of insulin secretion and signaling in a 12-week CUMS mouse model. This comorbidity may be due to chronic cytokine-mediated inflammatory responses, which characterized by activation of NLRP3 inflammasome and consequent IL-1β production, and induced by dysregulation of HPA axis. As an inhibitor of the NLRP3 inflammasome and antidiabetic drug of sulfonylureas, glyburide modulated not only depressive-like behavior, but also metabolic disturbance induced by chronic stress. We tend to attribute this effect mainly to the inhibition of NLRP3 inflammasome via suppressing its upstream molecule TXNIP, as coinciding with discoveries from Zhou et al. [42]. Furthermore, it is also confirmed that fluoxetine exerts an inhibitory effect on NLRP3 inflammasome possibly through downregulating TXNIP, which may help explain why fluoxetine can ameliorate inflammatory biomarkers [43]. Interestingly, fluoxetine can even regulate insulin signaling and secretion somehow.

Though many researchers believe cortisol dysregulation and chronic immune activation are the critical bidirectional links between stress, depression, and type 2 diabetes mellitus [11, 27], there is still a lack of adequate evidence and specific molecules which function as the key node. As Busillo et al. [29] elucidated, GC could sensitize the innate immune system by regulating NLRP3 inflammasome. The cleavage and maturation of pro-IL-1β caused by caspase-1 are largely mediated by inflammasome activation [44]. A great number of animal and human studies have demonstrated the role of IL-1β in facilitating β-cell dysfunction and IR [45]. Besides, Reich and colleagues [30] suggested that TXNIP was a mediator of GC-induced β-cell apoptosis. ROS-induced TXNIP overexpression could drive hepatic inflammation as well as lipid accumulation, diabetic retinopathy and diabetic nephropathy through activation of NLRP3 Inflammasome [46–48]. Apart from that, TXNIP was also explored in mediating CUMS-induced depression by activating inflammasome [49]. It seems that GC/ROS-TXNIP-NLRP3 pathway may be an alternative option for the aforementioned key nodes in comorbidity.

Metabolic tests composed of IPGTT, ITT, GSIS, and HOMA-IR in our research indicated that stressed mice got insulin-resistant phenotypes, but were not obvious in hyperglycemia. This is not exactly consistent with previous studies. For example, Pan et al. found that after a 12-week CUMS regimen, rats exhibited depression-like behavior and glucose intolerance. He reckoned that the signal cross-talk between hypothalamic corticotrophin-releasing factor (CRF) system and insulin might be impaired, while fluoxetine treatment adjusting that system potentially prevented or healed depression and comorbid diabetes [50]. Besides, similar CUMS-induced depressive co-morbid glucose intolerant phenotypes were also observed in some other studies and could be reversed by rosiglitazone, umbelliferone and curcumin [51–53]. In contrast, another recent work reported down-regulated blood glucose in depression mouse model [54]. Indeed, during the preclinical period of T2D, hyperinsulinemia and β-cell hyperplasia were often developed to compensate for insulin resistance [55]. On this basis, we would rather define it as “depression comorbid with prediabetes” in our present research, because decreases in islet insulin area, increased infiltrations of F4/80 positive inflammatory cell and disturbances of insulin secretion were observed simultaneously. That coincided with papers published before, which suggested F4/80 positive macrophages invaded diabetic islets [41, 56], CD68 positive macrophages could be found isolated or dispersed between adipocytes, throughout and around the pancreatic islets [57], as well as another report insisted that macrophages mediate β-cell loss in T2D [58].

Reversely, depressive-like behavior also showed up in mouse models of diabetes and other IR-related diseases. Ernst et al. [59] found that db/db mice exhibited molecular alterations which were usually seen in neurological disorders. Sharma with his colleagues [60] reported the occurrence of depression-, psychosis-like symptoms and anxiolytic behavior in db/db mouse strain. Moreover, it was demonstrated, in a brain-specific knock out of the insulin receptor mice model, that IR in the brain altered dopamine turnover and resulted in behavioral disorders [61]. Within our study, hippocampal insulin signaling abnormalities also appeared, but whether it would cause dopamine turnover dysfunction needed further confirmation. Besides, high-fat diet could induce anhedonia by activating the purinergic P2X7 receptor-NLRP3 inflammasome pathway [62, 63]. Even more, a clinical investigation of polycystic ovary syndrome (PCOS) found that HOMA-IR was significantly associated with the risk of depression [64]. Meanwhile, depression-like behavior was also observed in a dehydroepiandrosterone-induced PCOS mouse model [65]. In fact, the HOMA-IR index tended to increase in the stressed mice we studied.

Glyburide is the first identified compound that prevents NLRP3 inflammasome activation, but the exact mechanism of its pleiotropic effects remains obscure [32]. Zhou el al [42] reported that glyburide exerted its function by suppressing the induction of TXNIP. Controversially, Masters et al. [66] doubted the role of TXNIP in altering the effect of glyburide on the inflammasome. Regardless of the mechanisms, the efficiency of glyburide was solid. For example, it could not only attenuate blood-brain barrier disruption in brain injuries induced by trauma [67] and myocardial injury induced by lipopolysaccharides, but also prevent brain swelling [68]. However, results from Lahmann et al. [35] suggested that only little glyburide reached the central nervous system when given systemically. Therefore, we think that the anti-depressive and anti-IR effect of glyburide may be due to the inhibition of circulating inflammasomes, but its activity similar to peroxisome proliferator-activated receptor gamma (PPAR-γ) agonists cannot be excluded [69]. Anyhow, there exists a certain peripheral-cerebral communication which leads to quantities of diseases [70], which might help with the explanation. Nevertheless, chronic glyburide treatment in vivo may cause side effects including reversible loss of insulin secretory capacity due to β-cell hyperexcitability and other irreversible consequences [71, 72]. Apart from that, PPAR-γ agonists were also indicated to assist the treatment of major depression [73, 74], possibly owing to its anti-inflammatory effects, including inhibiting inflammasomes [75].

As for the complicated actions of fluoxetine used in this study, it, of course, requires some clarifications. As a classical antidepressant, fluoxetine has displayed sound effects of counteracting depressive-like behaviors. According to conventional wisdom, this might attribute to regulating reuptakes of synaptic 5-HT. Actually, fluoxetine inhibited activation of the inflammasome to a lesser extent than glyburide. However, it appeared that there was no difference between the two drugs for depressive phenotypes. In our view, this can be ascribed to their dissimilar anti-inflammatory properties, which led to the same destination. According to pre-clinical and clinical evidence, fluoxetine and other SSRIs (selective serotonin-reuptake inhibitors) could induce reductions in several proinflammatory cytokines [43], though not selectively. That is to say, the inhibitory effect on inflammasome may go through by-pass access, including the detrimental interactions between activation of inflammasomes and systemic inflammation [21]. Nevertheless, glyburide prevented mice from inflammasome-related inflammation specifically, which blocked the “final common pathway” leading to the development of depressive disorder. Similarly, the abovementioned mechanism can also be applied to illustrate the complex impacts on insulin signaling.

## Conclusions

To summarize, we put forward a presumption of CUMS inducing depressive like-behavior comorbid with insulin resistance through TXNIP-NLRP3 inflammasome pathway. According to the hypothesis, we indicated that antidiabetic glyburide could exert an effect on modulating such comorbidity via inhibiting NLRP3 inflammasome. It should be noted that this study mainly focused on the phenomena and limited underlying mechanisms, whereas the connection among neuroendocrine, immune and metabolic system was extraordinarily complicated. In order to further validate our postulations, more challengeable work needs to be completed. For example, the dosage effect of glyburide should also be confirmed, exact cell types in which NLRP3 inflammasomes activated shall be specified, and molecular pathways other than TXNIP-NLRP3 should be excluded or added. To date, we provided a potential therapeutic target for patients, especially those with comorbidity of depression and diabetes. It can also help to improve strategies for the prevention and treatment of diabetes and depression, respectively.

## Abbreviations

CRF: Corticotrophin-releasing factor
CUMS: Chronic unpredictable mild stress
DALYs: Disability-adjusted life-years
GTT: Glucose tolerance test
HOMA-IR: Homeostasis model of assessment for insulin resistance index
HPA: Hypothalamic-pituitary-adrenal
IL-1β: Interleukin-1β
IR: Insulin resistance
ITT: Insulin tolerance test
MetS: Metabolic syndrome
NLRP3: Nucleotide-binding domain, leucine-rich-containing family, pyrindomain-containing-3
OFT: Open field test
PCOS: Polycystic ovary syndrome
PPAR-γ: Peroxisome proliferator-activated receptor gamma
SPT: Sucrose preference test
T2D: Type 2 diabetes
TST: Tail suspension test
TXNIP: Thioredoxin-interacting protein
